# Orientation behavior of riparian long‐jawed orb weavers (*Tetragnatha elongata*) after displacement over water

**DOI:** 10.1002/ece3.7249

**Published:** 2021-02-09

**Authors:** Sidney J. Goedeker, Theresa E. Wrynn, Brian G. Gall

**Affiliations:** ^1^ Department of Biology Hanover College Hanover IN USA

**Keywords:** arachnid, navigation, riparian, spider

## Abstract

Many organisms possess remarkable abilities to orient and navigate within their environment to achieve goals. We examined the orientation behavior of a riparian spider, the Long‐Jawed Orb Weaver (*Tetragnatha elongata*), when displaced onto the surface of the water. When displaced, spiders move with alternating movements of the first three leg pairs while dragging the most posterior pair of legs behind them. In addition, spiders often perform a series of orientation behaviors consisting of concentric circles before ultimately choosing a path of travel directly toward the nearest point to land. While the number of orientation behaviors increased with increasing distance from shore, distance from shore had no effect on the direction of travel, which was significantly oriented toward the closest shoreline. These results indicate a complex ability to orient toward land when displaced onto water, possibly to decrease the amount of time on the surface of the water and thus decrease predation risk.

## Graphical abstract

Long‐jawed orb weavers orient and move toward the closest point of land when displaced from refugia onto the water's surface. At greater distances (3 m), spiders perform more orientation circles, but are still oriented toward the closest point of terrestrial habitat.

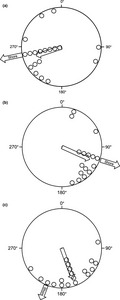

## 1 INTRODUCTION

Most organisms must move about their natural habitat to achieve goals, such as acquiring food and water, maintaining a territory, or finding a mate. While the complexity and importance of each specific goal or resource vary, the ability to identify oneself in physical space is critical to the animal's success or failure (Gaffin & Curry, 2020; Papi, 1992). While these movements can appear random and undirected, such as recently metamorphosed amphibians leaving a pond, this is rarely the case (e.g., Malmgren, 2002). Rather, complex mechanisms of orientation or navigation are frequently used to identify an organism's position within the space and make adjustments about where to travel.

The simplest mechanisms that guide animals through their environment include systematic searching, trail following, and path integration (Gaffin & Curry, 2020; Papi, 1992). In addition, more complex forms of goal acquisition include piloting and navigation (Gaffin & Curry, 2020; Papi, 1992). Organisms using piloting use familiar landmarks to orient and achieve a goal (Gaffin & Curry, 2020; Papi, 1992). This form of orientation is used by animals as diverse as insects and nonhuman and human primates (Dyer & Seeley, 1989; Epstein & Vass, 2014; Hauser, 2003; MacDonald et al., 2004). This mechanism contrasts with true navigation, which involves the use of a compass and map sense to calculate a goal direction (Gaffin & Curry, 2020; Goodenough et al., 2009; Papi, 1992). Organisms utilizing this form of navigation can compensate for displacement to obtain a goal (Phillips et al., 2006). Among animals, the cues involved in obtaining a compass bearing and navigating about their environment include polarized light (Marshall et al., 2019; Wehner, 1976), solar cues (Moore, 1980), and various celestial cues (Warren et al., 2019).

The cognitive requirements of the various mechanisms of orientation differ. Yet, many of the seemingly complex mechanisms have been documented among organisms that were previously thought to lack such abilities (i.e., invertebrates), and the previous decades have yielded explosive growth in studies evaluating orientation in these organisms (see references in Gaffin & Curry, 2020; Ortega‐Escobar, 2020; Pfeffer & Wolf, 2020; Warrant & Dacke, 2010). For example, the parasitic wasp *Hyposoter hotiola* uses visual landmarks to track host eggs and find potential plants that may contain those eggs (Van Nouhuys & Kaartinen, 2008). When navigating away from or toward their nest, desert ants (*Cataglyphis* sp.) may simultaneously use a combination of navigational mechanisms including systematic searching, landmarks, and path integration. These may be coupled with navigational cues (compass directions) with all systems aligned to create a navigational system, which allows the ant to maintain a trajectory toward a specific goal (Freas & Spetch, 2019; Pfeffer & Whittlinger, 2016; Wehner, 2003; Wehner et al., 2016). Even complex navigational abilities utilizing a map and compass sense can be found among arthropods (Pfeffer & Wolf, 2020). For example, dung beetles (*Scarabaeus lamarcki*) use a celestial compass as a cue to guide their navigations indicating map‐based navigation (Dacke et al., 2014).

Arachnida are a group of arthropods that may be particularly useful for understanding animal orientation and navigation given their relatively large size, flightless nature, and slow movements (see reviews by Gaffin & Curry, 2020; Ortega‐Escobar, 2020). Long‐jawed orb weavers (family: Tetragnathidae) are a group of spiders found across the Northern Hemisphere (Levi, 1981; Williams et al., 1995). Members of this genus are commonly found in habitats that are adjacent to both standing water and flowing fresh water (Gillespie, 1987). These nocturnal spiders build small webs parallel to the water surface (Gillespie, 1987) and prey upon emerging aquatic insects (Sanzone et al., 2003). Chemical analyses indicate they are an important part of aquatic food chains (Aiken & Coyle, 2000; Speir et al., 2014; Williams et al., 1995). These spiders are relatively fragile and desiccate quickly when not adjacent to freshwater (Gillespie, 1987). In the general course of working in riparian habitats, we have observed numerous instances of spiders being displaced onto the surface of the water from the surrounding vegetation. Despite a clear dependence on aquatic systems to provide structural habitat and food, the spiders’ ability to move on and return to their preferred habitat after such displacement is relatively unstudied. We conducted a series of studies to evaluate *T. elongata* navigational abilities on the surface of the water. First, spiders were displaced over water and an ethogram was generated to describe their responses on the water surface and shortly after contacting terrestrial habitat. Second, we placed spiders in both aquatic and terrestrial raceways and recorded their velocity. Finally, spiders were displaced at 0.75, 1.75, and 3.0 m away from shore and the direction of travel, relative to the shoreline, was recorded.

## 2 MATERIALS AND METHODS

### 2.1 Spider collection

**Figure 1:**
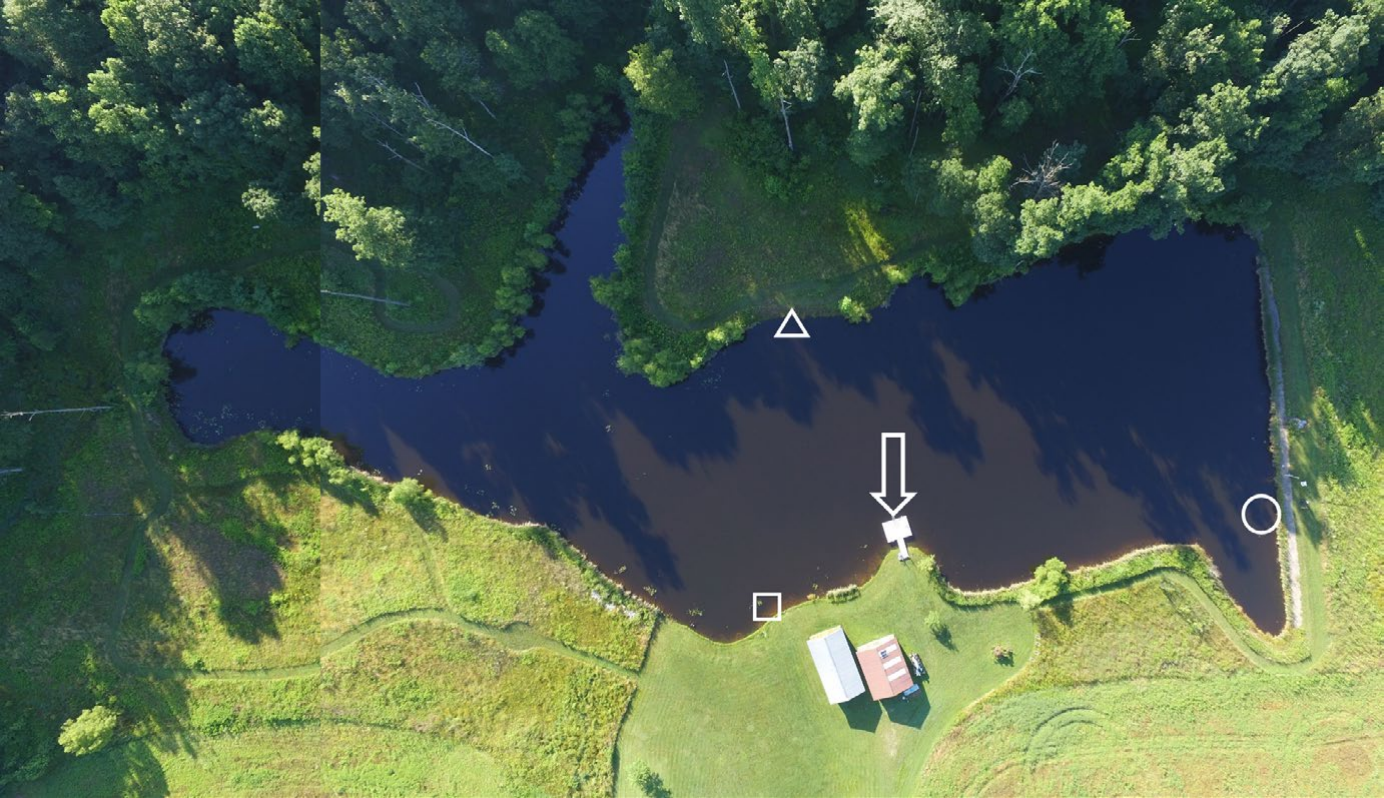
Aerial view of collection and test location where long‐jawed orb weavers (*Tetragnetha elongata*) were tested for orientation behavior after displacement. The collection site was a 3.6 × 5.0 m floating dock (arrow). Experimental trials were conducted at three different distances from shore including 0.75 m (square), 1.75 m (triangle), and 3.0 m (circle) at three different locations around the lake. Drop locations were selected to ensure approximately 90° difference between sites

Spiders were collected from a privately owned pond (1.2 ha; Figure 1) in Jefferson County, IN. Spiders were caught in opaque plastic containers and placed in an ice chest. The ice chest was transported to a climate‐controlled site 75 m from the collection site. During transport, the ice chest was gently spun several times in an attempt to disorient the spiders. Spiders were maintained in these containers until testing, which occurred approximately 2 hr after initial collection.

### 2.2 Orientation behavior

We conducted a series of observations on displaced spiders to thoroughly describe their behavior on the surface of the water. Observations were conducted on wind‐still evenings (approximately 20:00 hr) in June 2019. A spider was haphazardly selected and released onto the surface by gently shaking it out of the container. Spiders were released approximately 1 m from shore in an area of the pond near, but not consisting of, the collection site (collection location does not influence behavior when displaced onto the water; see below). Slow‐motion video was recorded on an iPhone 7 in slow‐motion camera mode for several individuals, and a second observer recorded detailed observations of the behavior of each spider. Observations that occurred during collection and testing were combined with the video and hand‐recorded observations, and an ethogram was constructed (Table 1).

**Table 1:** Ethogram of behaviors observed when collecting and displacing long‐jawed orb weavers (*Tetragnatha elongata*) away from the shoreline and onto the surface of the water

Name of behavior	Description
Orientation	Upon landing on the surface of the water, spider moves in a rapid clockwise or counterclockwise circle between 10 and 20 cm in diameter after which a path of travel is chosen
Silking	Spider releases silk strand (length undetermined) from abdomen that is caught in wind at upward angle. Abdomen becomes raised, legs extended, and leg movement ceases. Moves on the surface of the water in the direction of the wind, typically until contacting terrestrial habitat. Observed only when displaced over water. Occurred most frequently when wind appeared to impede forward movement toward shore.
Water walking	When displaced over water, spider alternates movement of front 6 legs. Two rear legs are extended outward behind the spider and are left motionless until contacting vegetation. Results in forward movement.
Ballooning	During process of silking, spider losses contact with surface of the water and becomes airborne. Occurs rarely, and only in the smallest spiders.
Elongation	Spiders will bring the front two leg pairs together anterior to the head and bring back two leg pairs together posterior to the body to form a stick‐like posture.

### 2.3 Spider velocity

The velocity of spiders on land (*N* = 20) and on water (*N* = 23) was assessed in the laboratory. Spiders were collected from the previously described pond (1.2 ha; Figure 1). To help prevent repeat testing of spiders, individuals for this study were collected from previously unsampled vegetated areas south and west of the initial collection site (minimum 20 m away). Spiders were placed in opaque containers and immediately transferred to Hanover College for testing. While unlikely, it is possible that spiders used in the velocity study had been tested previously.

To determine the velocity of spiders on land, a 17 × 125 cm raceway was constructed out of foam. On the side of the raceway, tick marks were placed every 10 cm. Freshly cut pieces of vegetation were laid on the horizontal surface of the raceway to provide a substrate. Large clumps of tall green vegetation were glued to the end of the raceway to provide a visual reference of shelter for the spider. A spider was then haphazardly selected, and the body length was recorded. It was then removed from the container by hand and placed at the start of the raceway. A timer was started, and without touching the spider, the observer used their hand to coax the spider down the raceway. The trial was terminated when the spider reached the vegetation at the end, went off the side, or stopped midway down the raceway. The distance between the starting location and end point was recorded. The time and distance traveled were then used to calculate the velocity with which spiders traveled. The spider was then placed back into their opaque container and later released at the site of collection.

The velocity of spiders on the surface of water was tested in plastic pools (100 × 20 cm deep). The pool was filled 1/3 full with tap water (23°C). A spider was haphazardly selected, and its body length was recorded. The spider was then placed in the middle of the pool, and as soon as they landed on the water, a timer was started. Once the spider reached the edge of the pool, the timer was stopped, and the spider was placed back into their holding container. Spiders generally traveled in a direct line to the edge of the pool. Any spider that did not travel in a direct path was removed from the analysis. The time and distance traveled were then used to calculate the velocity with which spiders traveled on the surface of water. We used linear regression to compare body length and relative velocity of spiders in terrestrial and aquatic raceways.

### 2.4 Orientation experiment

The orientation behavior of spiders after displacement over water was tested at 0.75 m (*N* = 19), 1.75 m (*N* = 16), and 3.0 m (*N* = 19) from the shoreline on the same pond in which spiders were collected. Trials were conducted on 26 June 2019 under wind‐still and clear skies. Three separate experimental sites were established corresponding to each of the three distances (Figure 1). Sites were selected such that the optimal direction to reach shore was different for each of the sites. Prior to experimentation, a brightly colored bobber was attached to fishing line and anchored to a lead weight. The weight was placed into the sediment of the pond such that the bobber was submerged below the surface of the water at the appropriate distance from shore. This bobber served as a visual cue for experimenters, ensuring the spiders were placed at the appropriate distance from shore across individual trials at each site. Once the bobbers were placed, a compass was used to determine the compass direction between the bobber and the closest point to land (0–360°).

At the start of a trial, an individual spider was removed from the opaque container and transferred into a square container glued to the end of a 3‐m plastic tube. To minimize the risk of influencing spider behavior, two observers dressed in camouflage clothing and face paint positioned themselves behind vegetation and lateral to the closest point to land from the release site; each observer was approximately 1 m from the spider's closest point of land. One observer moved the spider above the submerged bobber, inverted the container, and gently flexed the pipe to dislodge the spider onto the surface of the water. The direction of travel chosen by the spider was assessed using an imaginary circle (35 cm diameter) divided into eight equally sized sections of 45°. This diameter was selected to ensure that any orientation behaviors or initial adjustments to the preferred direction of travel were complete prior to recording the chosen direction; these adjustments almost always occurred within 10–15 cm of the release point after which a spider moves linearly. A second observer recorded the section of the circle in which the spider traveled. These same data were recorded by the first experimenter, and the median value of these observations was taken for each trial. In addition, the time it took for the spider to reach shore once released was also recorded. Once the spider reached shore, it was re‐caught and its body length was measured. Spiders were held until the completion of the experiment and were never retested. At the conclusion of the experiment, all spiders were released at the site of collection.

The circular data associated with each distance (0.75, 1.75, and 3.0 m) were assessed using V tests (Zar, 2010). This test is used to evaluate whether the mean angle of a set of observations is oriented in a predicted direction. In our case, the predicted direction was the closest point of terrestrial habitat from the release point. Briefly, the mean angle of all the observations at each distance is calculated (Zar, 2010). The value *r* is generated, which is a measure of dispersion of the observations; an *r*‐value closer to 1.0 indicates the observations are tightly clustered. A V test is then conducted, which generates the test statistic *u*. A nonsignificant *p*‐value indicates the observations are randomly oriented, whereas a significant *p*‐value indicates the observations are nonrandom and are oriented toward the closest terrestrial habitat. The time it took spiders to reach shore between the three distances was compared with an ANOVA followed by Tukey's post hoc comparisons. We also compared the number of orientation behaviors exhibited by spiders at each of the three distances with a contingency table (Zar, 2010). Finally, we conducted a Pearson correlation for each of the three distances with trial number and time to shore as the two variables to assess the role of trial order on the spiders’ orientation.

## 3 RESULTS

### 3.1 Orientation behavior

When displaced onto the surface of the water, spiders may exhibit one of several behaviors (Table 1). If close to shore (<1 m), and not oriented directly toward it, they often immediately turn toward the closest point of land and rapidly move on the surface of the water until they reach vegetation. However, moving to shore is often preceded by an orientation behavior (especially at distances > 1 m), whereby the spider rapidly moves on the surface in a ~ 15‐cm circle. A single orientation circle may be conducted or the spider may complete multiple (max: 8) orientation circles before choosing a path of travel. When walking on the surface of the water, spiders alternate movement of the front six legs only. The rear legs are left motionless and are drug behind the spider during forward movement. Finally, at any stage of the spiders return to shore, a strand of silk may be released from the abdomen. The abdomen is raised, the legs are extended, and all movement of the legs ceases. The spider is then pulled across the surface of the water in the direction the wind is blowing until reaching vegetation. In very light wind (<3.2 kph), extruding silk was observed more frequently when the spiders were displaced at greater distances. However, when the spider's optimal path of travel was hampered by stronger headwind (>3.2 kph), extruding silk became more common, and in some cases, spiders that were relatively close to shore (1–2 m) were observed to extrude silk and were pulled far across the surface of the pond to the opposite shore (>12 m).

### 3.2 Spider velocity

Spiders were approximately 10 times faster on water (mean ± standard deviation; 38.9 ± 20.1 cm/s) than on land (3.6 ± 1.3 cm/s). Body length was not related to the velocity of the spiders on land (*R* ^2^ = .12, *p* = .13) or on water (*R* ^2^ = .10, *p* = .15).

### 3.3 Orientation experiment

**Figure 2:**
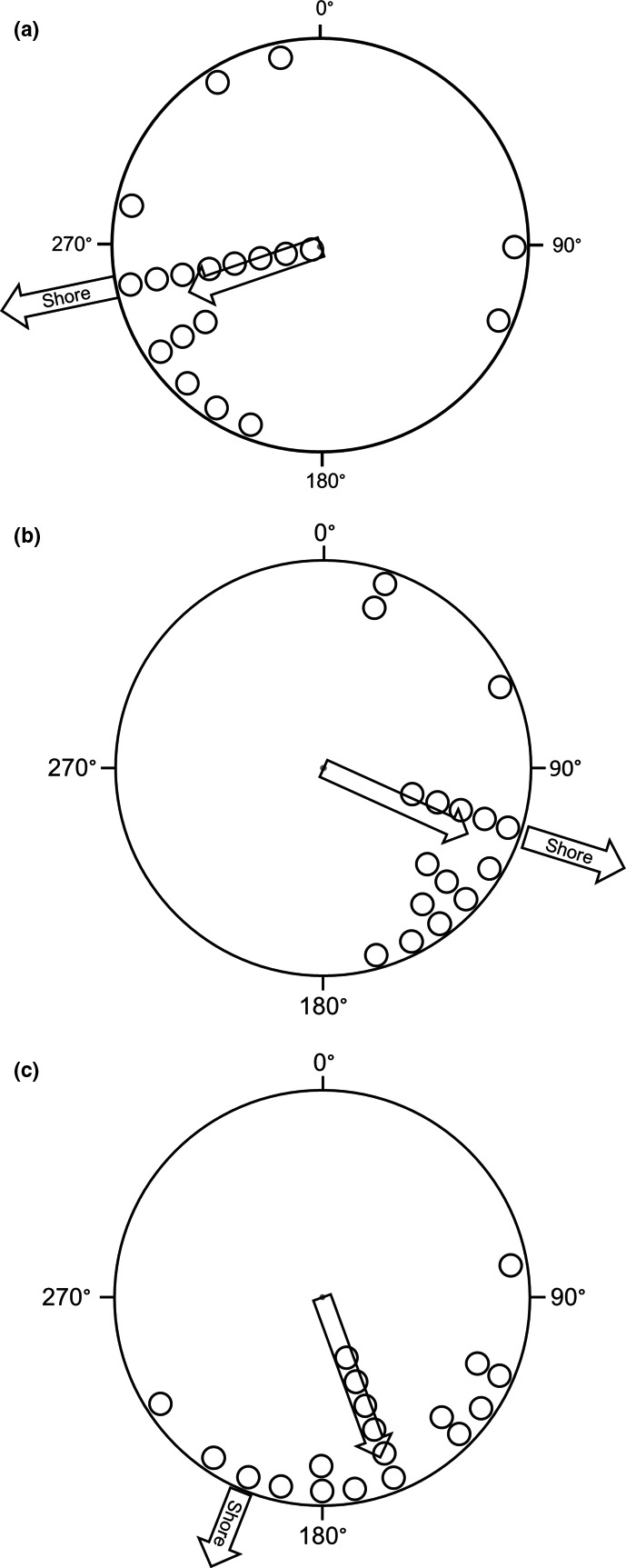
Orientation of long‐jawed orb weavers (*Tetragnetha elongata*) when displaced onto the surface of a lake at one of three different distances [0.75 m (a), 1.75 m (b), and 3.0 m (c)] from the closest shoreline. The compass angle of the shortest path to shore is indicated with the arrow outside the circle. Small open circles represent selections by an individual spider. The arrow inside each circle represents the mean angle of these observations with the length of the arrow representing *r*. Spiders at all distances were significantly oriented toward the closest shoreline (*p* ≤ .0005)

**Figure 3:**
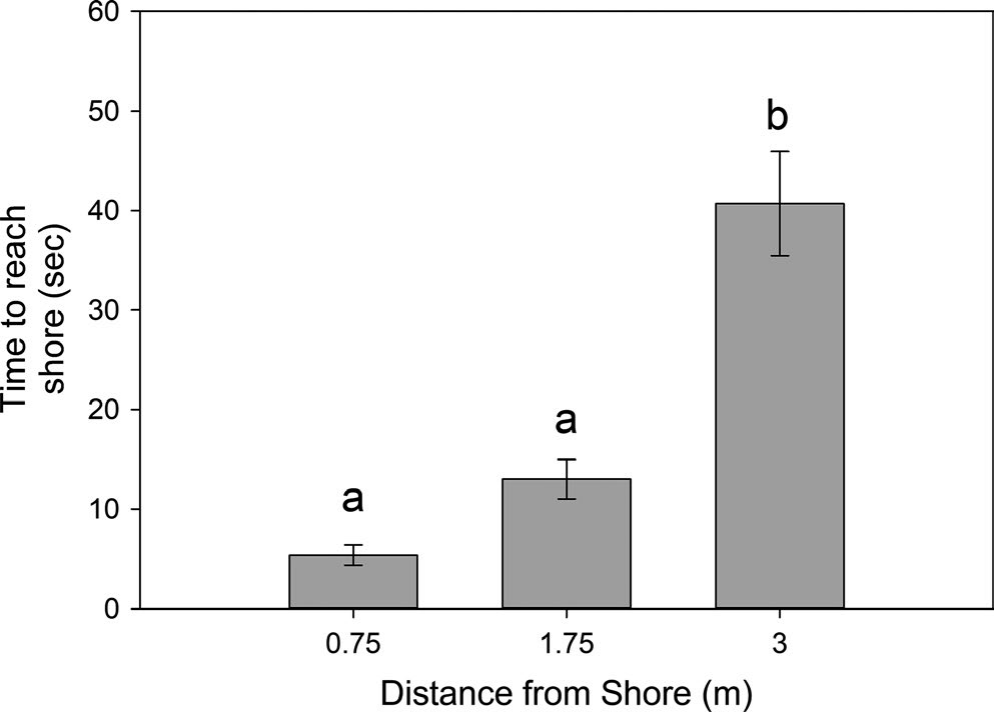
Mean (±*SE*) time to reach shore by long‐jawed orb weavers (*Tetragnetha elongata*) when displaced over water at three different distances from the shoreline. Spiders displaced 3.0 m took significantly longer to reach shore than spiders placed 0.75 or 1.75 m (*F*_[49,2]_ = 29.2, *p* < .001). Different letters indicate significant differences between groups (*p* < .001)

The mean angle chosen by spiders displaced 0.75 m from the shoreline was significantly oriented in the predicted direction of the nearest terrestrial habitat (*r* = .66, *p* < .0005; Figure 3). Similarly, when displaced 1.75 and 3.0 m from shore, the mean angle of the spiders was significantly oriented toward the predicted angle of the closest shoreline (1.75 m: *r* = .78, *p* = .0005; 3.0 m: *r* = .81, *p* < .0005; Figure 2).

Spiders took significantly longer to reach the shoreline at 3 m, compared with 0.75 and 1.75 m (*F*_[2,49]_ = 29.2, *p* < .001; Figure 3). In addition, spiders displaced 3 m from shore exhibited more orientation behaviors than spiders displaced 0.75 m or 1.75 m (*χ* ^2^ = 9.8, *p* = .007). Correlation analyses found no relationship between the sequence in which spiders were tested and the time they took to reach shoreline or number of orientation behaviors for any of the three distances (all *p* > .25), suggesting there is no relationship between trial order and the time to reach shore.

## 4 DISCUSSION

When displaced over water, long‐jawed orb weavers can differentiate shoreline from open water and orient themselves toward terrestrial habitat leading to rapid zonal recovery. These spiders either take an immediate and direct path to shore or perform one or more circular orientation behaviors before choosing a relatively linear path of travel. Spiders were robustly (*r* = .66–.81) clustered toward the closest shoreline, despite experimental drop locations with shoreline angled 90° or greater relative to their collection location. These results indicate that site fidelity is less important than minimizing time away from preferred habitat and is surprising given previous research on displacement in spiders (Morse, 2002; Papi, 1955; Tongiorgi, 1959). For example, wolf spiders (family Lycosidae) that are displaced to the opposite side of a stream actively return to the home side after release (Papi, 1955). This occurred under clear skies, but under overcast skies the spiders sought the closest shoreline (Papi, 1955). Research suggests that these spiders use a combination of visual cues including sun compass, polarized light, and landmarks to orient (Papi, 1955; Tongiorgi, 1959), with further research identifying the particular subset of eyes involved in Lycosid orientation (Magni et al., 1964; Ortega‐Escobar, 2006; Papi, 1955).

The ability to orient after displacement is not unique to spiders and has been documented in numerous arthropods including ants, bees, and wasps (Schöne, 1996; Ugolini, 1987; Wehner & Srinivasan, 1981), dung beetles (Baird et al., 2012), and butterflies (Srygley et al., 2006). However, this ability is likely particularly important in species occupying riparian habitats (e.g., lakeshores and seashore) given the unpredictable and rapidly changing nature of these regions (Herrnkind, 1972). In these cases, the ability to orient is likely linked to relative risk induced within the nonpreferred habitat (Lambeets & Bonte, 2009). For example, sand hoppers (*Talltrus* sp.) spend the entirety of their life in the featureless sandy zone between the terrestrial environment and open sea. These species are exposed to extreme desiccation risk in arid inland habitats, but simultaneously drown if submerged in water. This narrow range of optimal habitat, combined with the need to move inland at night to feed while remaining moist (but not submerged) during the day, has led to extensive orientation and zonal recovery abilities in these species (see review by Scapini, 2006). Even at the edge of the lentic habitat long‐jawed orb weavers occupy, strong winds, heavy rain, and rapidly rising water levels can dramatically alter the habitat and potentially displace them into predator‐rich areas (Bates et al., 2006). During collection, we routinely observed spiders abandon refugia for the water surface, which was always followed by rapid orientation and zonal recovery. In these cases, identification and collection of the spiders were rare. We hypothesize that refuge abandonment may be a common mechanism to reduce the risk of predation, possibly from insectivorous birds such as blackbirds or other thrushes (Family: Turdidae), which we routinely observe feeding in this habitat. This is followed by rapid orientation and zonal recovery once on the water surface, which likely evolved to decrease predation risk from fishes, which actively prey on surface‐bound insects (Mehner et al., 2005).

Surprisingly, the distance (0.75, 1.75, and 3.0 m) at which spiders were dropped from shore had no effect on their ability to distinguish shoreline and orient themselves to the closest point of land. While their general path of travel was correctly oriented, the spiders did exhibit a significantly greater number of orientation behaviors with increasing distance, exacerbating the time it took them to reach shore after being displaced at these longer distances. These results suggest that *T. elongata* spiders require more extensive external inputs from the surrounding environment to accurately determine an effective path of travel at some distance beyond 1.75 m. While the stimuli used by these spiders to assess their environment and orient are unknown, arthropods have been documented to utilize a variety of media to aid in navigation. For example, dung beetles and sand hoppers use a sun compass (Baird et al., 2012; see review by Scapini, 2006) and snapping shrimp use visual cues to orient (Huang et al., 2005). In some spiders, visual references such as the sun and polarized light may be critical to orientation (Görner, 1962; Ortega‐Escobar & Munoz‐Cueva, 1999; Papi, 1955) with specific subsets of eyes being involved with processing different sources of visual information (see review by Morehouse et al., 2017). Yet in other species, *Cupiennius salei*, ablating the eyes has little effect on navigation (Barth & Seyfarth, 1971; Seyfarth & Barth, 1972). In these cases, numerous small slits located on the legs, known as lyriform organs, likely serve to provide air current or olfactory information that permits the animals to navigate in the absence of vision (Schmid, 2014; Wiegmann et al., 2019; Young et al., 2016). All the trials in our study were conducted under clear skies when sun compass and polarized light cues should have been available. Yet, Long‐Jawed orb weavers always oriented toward the closest point of land, and therefore, we hypothesize that *T. elongata* utilize visual landmarks produced by the shoreline to identify suitable habitat. Nevertheless, further research is necessary to determine the mechanisms by which these spiders orient on the surface of the water.

The speed of the long‐jawed orb weavers over water was substantially greater to movement on land and is consistent with previous studies on the relative movements of this family on land and water (Suter et al., 2003). These spiders utilize different gaits on land versus water and are significantly faster on the water's surface than numerous other spiders (Suter et al., 2003). This could be due to numerous factors including the relative predation risk between the sites or the complex structure of the terrestrial environment compared with the homogenous nature of the water's surface. Many aquatic insects and spiders have evolved water walking in such a way that different species have developed different gaits (walking, rowing, etc.) to increase their speed across the water (Hu & Bush, 2010). In addition, setae on the limbs help spiders and insects stay above water and can help influence the velocities that can be attained by these species (Hu & Bush, 2010). Relative to other spiders, the Tetragnathids have evolved a specialized and efficient gait producing a faster velocity on the surface of water (Suter et al., 2003).

The results of this study show that long‐jawed orb weavers have the ability to orient and navigate toward preferred habitat when displaced on the surface of the water. In addition, distance at which they were displaced had no effect on their ability to locate the closest shoreline. These results combined with circular orientation behaviors performed at greater distances from shoreline indicate that some type of visual cue may be necessary to find shoreline. While these spiders are highly dependent on water for food and the riparian habitat in which they reside, the ability to identify shore and the preference for zonal recovery over site fidelity suggests that pressure to avoid the water surface, possibly due to predation, is intense.

## CONFLICT OF INTEREST

All authors declare no conflict of interest.

## AUTHOR CONTRIBUTIONS

**Sidney Goedeker:** Conceptualization (equal); Data curation (equal); Formal analysis (equal); Methodology (equal); Writing‐original draft (equal). **Brian Gall:** Conceptualization (equal); Data curation (equal); Formal analysis (equal); Investigation (equal); Methodology (equal); Project administration (equal); Resources (equal); Software (equal); Supervision (equal); Validation (equal); Visualization (equal); Writing‐original draft (equal); Writing‐review & editing (equal).

## Supporting information

Video S1

## Data Availability

These data are available in the Dryad database under the following link: https://doi.org/10.5061/dryad.z34tmpgc9
